# Rehabilitation of Congenitally Missing Bilateral Incisors With the Maryland Bridge: A Case Report

**DOI:** 10.7759/cureus.58349

**Published:** 2024-04-15

**Authors:** Ekta M Kanojia, Anjali Bhoyar, Surekha A Dubey, Seema Sathe, Sheetal R Khubchandani

**Affiliations:** 1 Prosthodontics and Crown and Bridges, Sharad Pawar Dental College and Hospital, Wardha, IND; 2 Prosthodontics, Sharad Pawar Dental College and Hospital, Wardha, IND

**Keywords:** minimally invasive procedures, electrolytic etching, micromechanical retention, resin-bonded bridge, congenitally missing bilateral incisors, maryland bridge

## Abstract

Congenital partial hypodontia is a commonly encountered disorder, presenting a challenge for adolescents seeking treatment, as existing options often come with drawbacks. Among these options, the Maryland Bridge stands out for its accessibility and notable benefits in terms of both strength and aesthetics. This article explores the merits of this treatment modality, supported by a detailed case study demonstrating its successful application. An 18-year-old patient was referred to our hospital with a complaint of missing bilateral maxillary incisors. Upon clinical examination, it became apparent that the orthodontic treatment was done and exhibited the absence of bilateral incisors in the upper arch. Subsequent diagnosis confirmed congenital partial hypodontia. To address the missing teeth, a treatment plan centered around the use of a Maryland Bridge was devised. One of the persistent challenges faced by restorative dentists is devising solutions for congenitally missing lateral incisors. Despite the availability of numerous therapeutic alternatives, none are without their limitations. However, the outcome of the rehabilitation in this case proved to be notably aesthetically pleasing, effectively fulfilling the intended purpose. As a result, this article advocates for the Maryland Bridge as a viable option for patients facing similar dental challenges.

## Introduction

The fundamental design of a resin-bonded bridge involves a metal framework that supports the artificial tooth, with the palatal surface of one or more abutment teeth being connected to this framework using composite resin and the acid-etch technique [1]. This technique involves applying a mild acidic solution to the enamel surface of the teeth to create a roughened texture, which allows the resin to adhere more effectively. The composite resin acts as the bonding agent between the metal framework and the natural teeth, providing stability to the bridge [2].

Various methods can be used to attach the composite resin to the metal framework, depending on factors such as the type of material used and the specific requirements of the case [3]. Despite the possibility of de-bonding (the bridge coming loose from the abutment teeth), resin-bonded bridges are generally successful in restoring missing teeth in the aesthetic region of the mouth. They are favored for their ability to achieve good survival rates and for offering minimally invasive procedures that can be performed within a general dental setting [4]. When rehabilitating a patient with a resin-bonded bridge, it is necessary to prepare the adjacent teeth to ensure proper retention and stability of the bridge. This may involve creating retentive vertical grooves and wing-shaped preparations on the abutment teeth, which help secure the bridge in place [5].

Implant placement is usually deferred until there is conclusive evidence of growth completion, especially in younger patients. This evidence may include cephalometric radiographs indicating that skeletal growth has ceased. Implant-supported restoration is widely regarded as the most substantial, aesthetically pleasing available option, based on studies conducted over prolonged periods [6]. It is important to note that resin-bonded bridges are not a no-prep solution; they require thorough treatment planning and technical expertise to ensure successful outcomes. Preparation of abutments for resin-bonded fixed partial denture (FPD) demands meticulous attention to detail, including appropriate alterations to the enamel to establish a distinct incisal-gingival path of insertion and resistance form [7].

This clinical report outlines a conservative technique for replacing missing upper lateral incisors using a Maryland Bridge, which is a specific type of resin-bonded fixed partial prosthesis. This technique involves careful preparation of the adjacent teeth and precise bonding of the bridge to achieve optimal aesthetic and functional results.

## Case presentation

**Figure 1:**
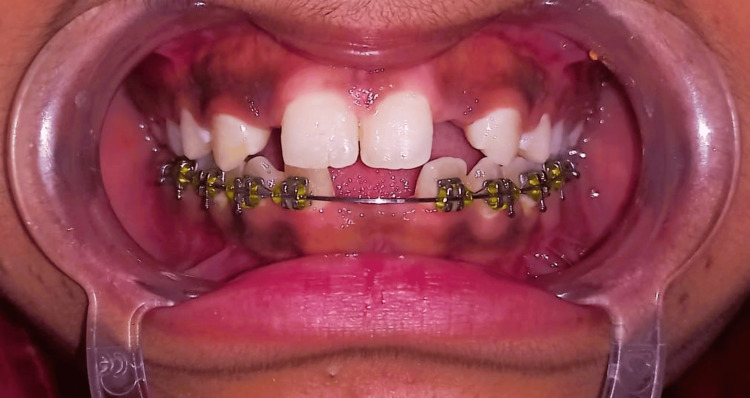
Pre-operative frontal image

**Figure 2:**
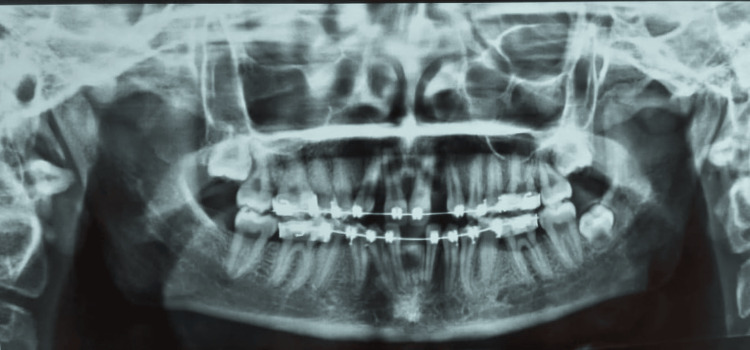
OPG image OPG: orthopantomogram

An 18-year-old female visited the Department of Prosthodontics at Sharad Pawar Dental College and Hospital, DMIHER (Sawangi) Wardha, with concerns about missing upper front teeth and unhappiness with her smile. Examination was done, and it was noted that she was missing both lateral incisors due to congenital etiology but had overall good oral health, as evidenced by her dental condition shown in Figure 1. Also in the orthopantomogram (OPG) image, there is a congenital absence of lateral incisors depicted in Figure 2.

**Figure 3:**
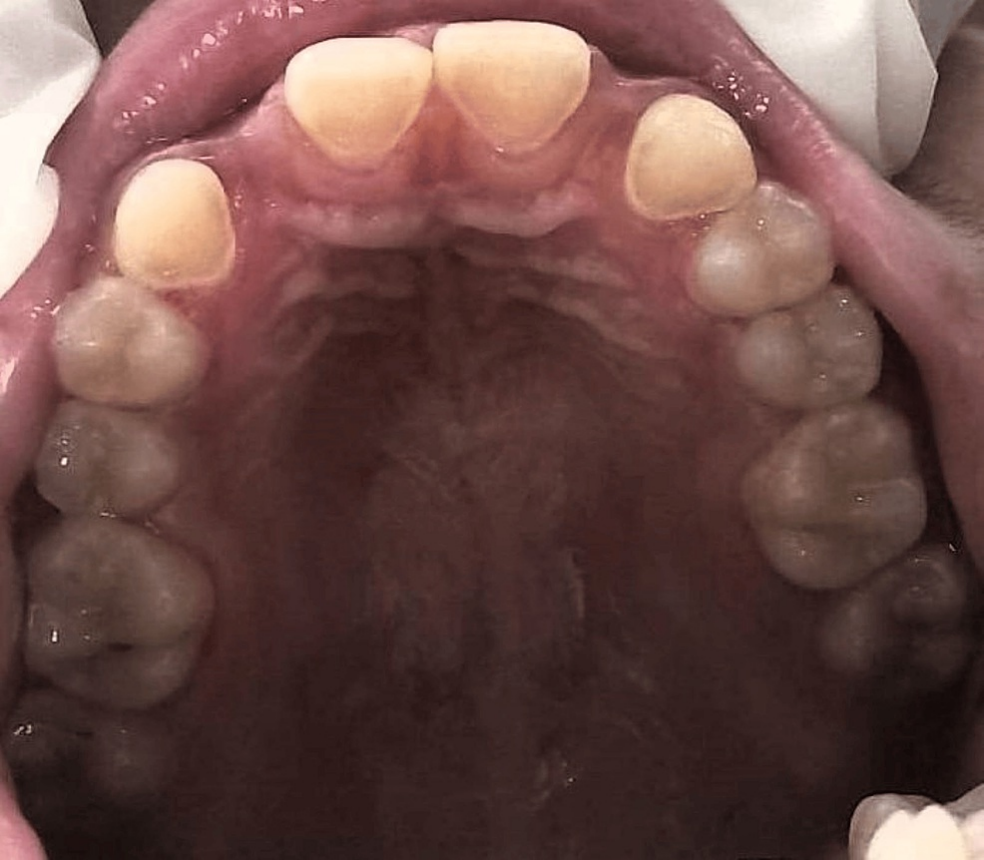
Tooth preparations on maxillary central incisors and canine tooth on both the quadrant

The patient had previously undergone orthodontic treatment for two years to address crowding in her lower jaw. During this time, she wore a prosthesis in place of the missing lateral incisors, which were supported by orthodontic braces. Following the completion of her orthodontic treatment, the patient expressed a desire to replace her missing teeth with a long-lasting and aesthetically pleasing solution. However, due to her age and ongoing physical development, implant placement was not deemed suitable at that time. After weighing her options, the patient opted for a Procera Maryland Bridge as a temporary solution. This choice was made based on its ability to provide both durability and aesthetic appeal. The patient underwent a psychological assessment, which indicated sound mental health and realistic expectations regarding the treatment outcomes. The procedure involved minimal tooth preparation, focusing only on the lingual surfaces of the abutment teeth. This approach helps preserve as much natural tooth structure as possible. Detailed tooth preparation steps include wing-shaped preparations made on the canines, involving careful shaping of the cingulum area using specialized dental instruments. Vertical grooves were created on the central incisors to enhance the bonding surface for the bridge. Special attention was paid to ensuring that the preparation margins did not extend beyond certain anatomical landmarks to maintain the integrity of the teeth as depicted in Figure 3.

**Figure 4:**
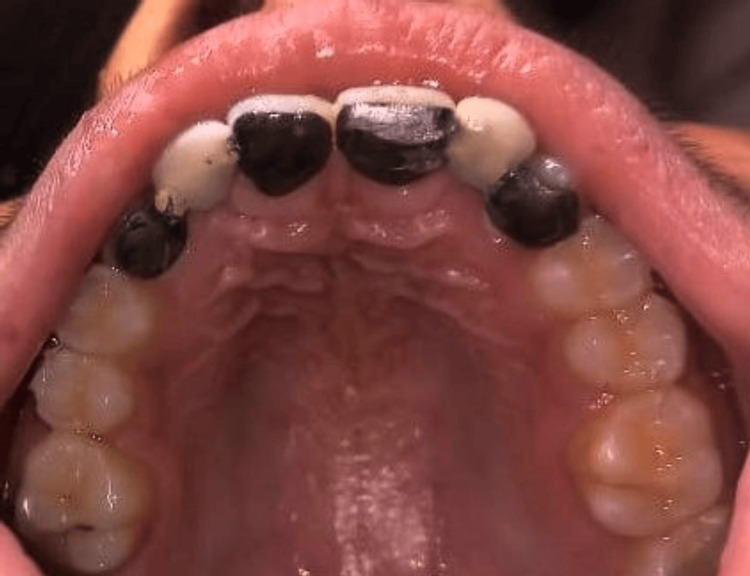
Post-operative occlusal image after cementation of the prosthesis

An impression of the prepared teeth was taken using advanced silicone impression material, which was then sent to the laboratory for fabrication of the bridge. The bridge was crafted from a non-precious alloy, and shade selection was meticulously done to match the patient's natural teeth. The completed bridge was securely bonded to the prepared teeth using universal self-etch resin cement. This step ensures a strong and long-lasting attachment of the bridge to the abutment teeth as shown in Figure 4.

**Figure 5:**
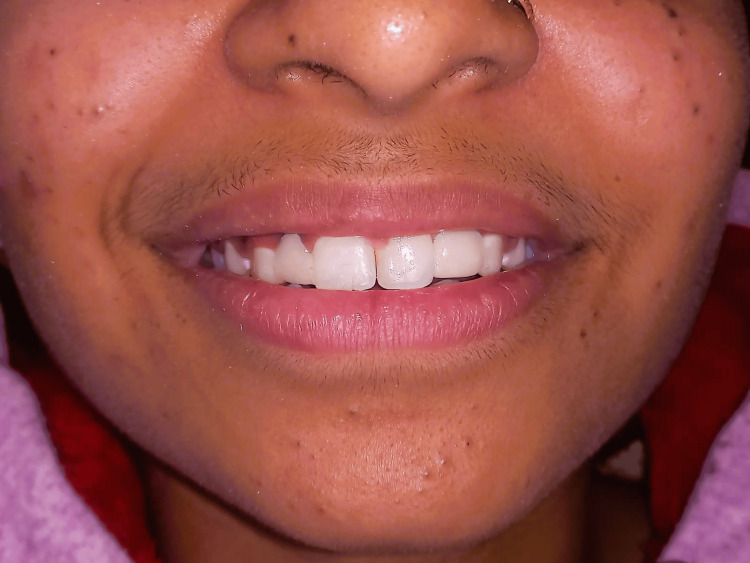
Post-operative frontal image

The patient's bite was carefully assessed at various jaw positions to ensure proper alignment and the absence of any interference from the newly placed bridge as shown in Figure 5.

Detailed instructions were provided to the patient regarding post-cementation care, including dietary restrictions, oral hygiene practices, and steps to take in case of any issues with the bridge. The patient attended follow-up appointments to assess the stability and functionality of the bridge. It was confirmed that there were no issues with the bridge, and the patient was satisfied with the aesthetic outcome of the procedure.

## Discussion

The resin-bonded Maryland Bridge stands out as a preferred option due to its minimally invasive approach, which is characterized by its ability to preserve a more natural tooth structure compared to traditional bridges. This conservative nature makes it particularly advantageous for younger patients with congenital partial hypodontia, a condition where individuals are born with one or more missing teeth [8]. By minimizing the need for extensive tooth preparation, the Maryland Bridge offers a solution that not only addresses the aesthetic and functional concerns associated with missing teeth but also ensures the long-term health and integrity of the remaining dental structures [9].

Extensive research has underscored the efficacy of Maryland Bridges in addressing congenital partial hypodontia. Studies have consistently demonstrated positive outcomes in terms of both aesthetics and functionality. This empirical evidence provides reassurance to both patients and practitioners regarding the reliability and effectiveness of this treatment modality [10]. Key factors contribute to the success of Maryland Bridges. Electrolytic etching, for instance, plays a crucial role in preparing the tooth surface for bonding by creating micropores. These micropores enhance the adhesion of the resin cement, thereby ensuring a durable bond between the bridge and the tooth. Additionally, micromechanical bonding, facilitated by microscopic irregularities on the tooth surface created through etching, further improves the bridge's retention [11].

Precise construction and fitting are also paramount. Accurate fabrication and placement of the bridge are essential for achieving optimal results in terms of both function and aesthetics. This precision ensures proper alignment and fit, minimizing the risk of complications such as discomfort or compromised functionality [12]. The advantages of Maryland Bridges extend beyond their conservative approach. Reduced pulpal damage is a significant benefit, as minimal tooth preparation minimizes the risk of trauma to the dental pulp, especially in young patients with large pulp chambers. Furthermore, advancements in resin cement have led to improved bond strength between the bridge and the tooth surface, ensuring better retention over time [13].

The use of non-precious alloys further enhances the performance of Maryland Bridges. These alloys are preferred for their ability to undergo micromechanical retention, which contributes to the overall stability and longevity of the restoration. Despite its numerous benefits, the successful placement of a Maryland Bridge requires careful consideration of various factors. Proper case and abutment selection are crucial, as they directly influence the bridge's fit, function, and longevity [14]. Additionally, bridge design considerations, such as retainer wing coverage and vertical depth grooves, play a significant role in determining stability and aesthetics. Technical precision during fabrication and placement cannot be overstated. Attention to detail ensures that the bridge integrates seamlessly with the natural dentition, providing both functional and aesthetic harmony. However, challenges exist, including the technique-sensitive nature of the placement and the visibility of the metal retainer, particularly in the anterior teeth region. Addressing these challenges requires careful planning and execution [15]. Techniques to minimize retainer visibility and prevent caries around bridge margins are essential. Meticulous sealing of the prosthesis and maintaining optimal oral hygiene are critical in this regard [16]. In conclusion, the resin-bonded Maryland Bridge offers a conservative yet effective solution for rehabilitating congenital partial hypodontia. Its success hinges on meticulous attention to various factors, including proper case selection, precise fabrication and placement, and preventive measures to ensure long-term efficacy and patient satisfaction [17].

Limitation

The Maryland Bridge can debond partially or completely, which means that it detaches from the abutment teeth. This can lead to issues such as discomfort, compromised aesthetics, and functional limitations. Furthermore, in cases of partial debonding, where only a portion of the bridge becomes detached, there is a risk of food becoming lodged between the bridge and the abutment teeth. This can create an environment conducive to the development of secondary caries, which are cavities that form adjacent to existing dental restorations.

Despite these possible limitations, Maryland Bridges remains a highly feasible treatment option, particularly for improving dental aesthetics and delivering good functional and cosmetic results.

## Conclusions

Resin-bonded bridges provide substantial benefits in terms of replacing lost teeth while also restoring dental function and aesthetics. These bridges are particularly effective for addressing small spans where minimal tooth preparation is desired. However, their success relies heavily on thorough patient assessment and the careful execution of clinical procedures.

Given the advantages of resin-bonded bridges and their potential to deliver favorable outcomes, dental professionals need to consider them more frequently as the restoration of choice, especially in cases where preserving tooth structure and achieving aesthetic results are paramount. By prioritizing patient evaluation and employing precise clinical techniques, resin-bonded bridges can serve as an excellent option for addressing various dental concerns while maximizing patient satisfaction.
